# Comparing and contrasting clinical consensus and guidelines for anal intraepithelial neoplasia in different geographical regions

**DOI:** 10.1007/s13304-021-01156-7

**Published:** 2021-09-04

**Authors:** Danielle R. L. Brogden, Micol E. E. Lupi, Oliver J. Warren, Christos Kontovounisios, Sarah C. Mills

**Affiliations:** 1 Chelsea and Westminster Hospitals NHS Foundation Trust, London, UK; 2 Imperial College London, London, UK

**Keywords:** Anal squamous cell carcinoma, HIV, Human papillomavirus, Anal intraepithelial neoplasia, Dysplasia

## Abstract

Anal Squamous Cell Carcinoma (ASCC) is an uncommon cancer with a recognised precursor Anal Intraepithelial Neoplasia (AIN). Although there are consistent evidence-based guidelines for the management of ASCC, historically this has not been the case for AIN and as a result there have been geographical variations in the recommendations for the treatment of AIN. More recently there have been updates in the literature to the recommendations for the management of AIN. To assess whether we are now closer to achieving an international consensus, we have completed a systematic scoping review of available guidelines for the screening, treatment and follow-up of AIN as a precursor to ASCC. MEDLINE and EMBASE were systematically searched for available clinical guidelines endorsed by a recognised clinical society that included recommendations on either the screening, treatment or follow-up of AIN. Nine clinical guidelines from three geographical areas were included. The most recent guidelines agreed that screening for AIN in high-risk patients and follow-up after treatment was necessary but there was less consensus on the modality of screening. Six Guidelines recommended the treatment of high-grade AIN and four guidelines describe a follow-up protocol of patients diagnosed with AIN. There appears to be increasing consensus on the treatment and follow-up of patients despite a poor evidence base. There is still significant discrepancy in guidance on the method to identify patients at risk of ASCC and AIN despite consensus between geographical regions on which patient subgroups are at the highest risk.

## Introduction

Anal squamous cell carcinoma (ASCC) is an uncommon Human Papillomavirus (HPV) linked cancer with an incidence rate of between 1–2 per 100,000 per year. It has a dysplastic precursor Anal Intraepithelial Neoplasia (AIN).

HPV virus is a non-enveloped double-stranded DNA virus with over 100 known genotypes with approximately 30 genotypes that can be spread through sexual contact [1]. Up to 95% of ASCC’s have high-risk oncogenic HPV infections [2, 3] with the most common high-risk genotype being HPV 16 [3–5].

Risk factors for the development of ASCC include HIV, receptive anal intercourse, previous HPV related dysplasias and malignancies at neighbouring sites as well as any clinical state which would inhibit easy clearance of an HPV infections such as long-term use of steroids in transplant patients.

Although there are comprehensive evidence-based guidelines for the treatment of Anal Squamous Cell Carcinoma [6–9] which are largely consistent with each other [10] this is not also the case for guidelines dealing with the treatment of AIN.

In the case of AIN, the development of standardised guidelines has been hampered by small study sizes and poor study design. There is also the difficultly that different geographical areas use different nomenclature for defining AIN so results of small studies from different countries are not easily collated into a meta-analysis for better evidential results. Attempts to improve this situation have been comprehensively undertaken by the consensus achieved by the Lower Anogenital Squamous Terminology (LAST) project guidelines that clearly define AIN to either high-grade squamous intraepithelial lesion (HSIL) or low-grade squamous intraepithelial lesion (LSIL) using the biomarker p16, associated with high-grade lesions [11, 12], as a differentiating factor [13]. Despite this consensus, the nomenclature is not always used.

Historically, the lack of good quality evidence has meant that societies in different geographical regions have different clinical recommendations for best practice. This can be based on clinician preferences as well as available health care resources in their regions.

Over the last few years, guidelines in different geographical areas have been published which appear to recommend different approaches to the previous management of AIN. To assess whether we are now closer to achieving an international consensus, we have completed a thorough and up to date systematic scoping review of available guidelines for the screening, treatment and follow-up of AIN as a precursor to ASCC.

## Method

This is a systematic scoping review completed following the Reporting Items for Systematic Reviews and Meta-Analyses Extension for Scoping Reviews (PRISMA-SCR) Statement.[14–16].

### Search strategies and data sources

MEDLINE and EMBASE were searched from database inception to 29th November 2020 using our search strategy [(“Anal Squamous Cell Carcinoma” OR “Anal Cancer” OR “Anal Intraepithelial Neoplasia”) AND (“guideline$” OR “consensus” OR “management” OR “screening”)]. References lists of included and key papers were also searched as well as relevant clinical societies webpages and their associated journals.

The titles and abstracts obtained from the search were systematically compared to our inclusion criteria for relevant full papers to be read. If on reading, the full papers met the inclusion criteria they were included in the review. DRLB and MEEL performed separate independent data searches and compared the final full papers to be included. If there was a discrepancy identified a third party within the research group was asked to give an independent opinion. Duplicate papers were excluded on abstract review.

### Inclusion criteria

To be included in the scoping review, the guidelines must be published and endorsed by a clinical society or an expert taskforce in the English language and give advice or recommendations on either the screening, treatment or follow-up of AIN.

### Exclusion criteria

Guidelines or recommendations given by papers based on a single centre experience or a case series were excluded.

### Levels of evidence

Where the guideline has already used a tool to state the level of evidence behind their recommendations, this will be stated with the recommendation. If the guideline had not completed this exercise the authors assessed the evidence in the study referred to in the guideline and classified them by the GRADE recommendations [17]. If a recommendation is given without stating evidential support, this will be stated as opinion next to recommendation.

## Results

As a wide literature search was required, a high number of titles were first identified (*n* = 2804). This was eventually reduced to nine clinical guidelines that included recommendations for the screening, management, and treatment of AIN (Table 1). 4 further guidelines were identified that were superseded by more recent up to date guidelines.

**Table 1:** Summary of recommendations by organised by geographical area of current included guidelines

Country of origin and clinical society	Guideline level of evidence/grading system	Screening	Treatment	Follow-up
United States of America				
American Society of Colon and Rectal Surgeons (ASCRS)	Stewart et al. [7] GRADE Recommendation System	Anal Cytology can be used in high-risk populations as part of a directed screening programme but not recommended as a universal screening technique (2B) HPV genotyping can be used as an adjunct within the screening programme (2B) High resolution anoscopy can be used as a screening technique but only when used by experienced practitioners (2B)	Topical treatments such as Imiquimod, 5-Fluorouracil, Cidofovir and Trichloroacetic acid can be used to treat low-grade and high-grade AIN. (2B) Ablative treatments can be used for high-grade AIN (2B) HPV vaccination is not recommended as a form of secondary prevention of AIN (2A)	Patients treated for AIN may be observed without regular cytology, HPV testing or HRA but any visible or palpable disease should be treated (2C)
National Comprehensive Cancer Network (NCCN): People Living with HIV	Reid et al. [25] GRADE Recommendation System	No recommendations for routine screening but accepts that HIV specialists do screen using varying methods and at different frequencies High resolution anoscopy should be performed if high-grade disease is identified (1C)^a^	Topical therapies, ablative therapies and surgical excision are safe and offer short term efficacy (1C)^a^ Electrocautery better choice of therapy in men who have receptive anal intercourse (1B)^a^	No recommendations
New York State Department of Health AIDS Institute (NYSDOH)	New York State Department Levels of Evidence and Recommendation System Medical Care Criteria Committee and Brown [26]	Annual history (2A), digital rectal examination and anal cytology (3A) in HIV positive patients ≥ 35 years Refer for high resolution anoscopy if anal cytology is abnormal (2A) Cervical cytology if abnormal anal cytology (3A) Annual high resolution anoscopy if high-risk HPV genotypes (GRADE 1C)^a^	AIN1—surveillance only (3A) AIN2/3—recommends treatment (no preference stated) (3A)	AIN1—high resolution anoscopy in 1 year (3A) AIN2/3—high resolution anoscopy 6 months after treatment (3A)
United Kingdom				
Association of Coloproctology of Great Britain and Ireland (ACPGBI)	Geh et al. [6] ASGBI Guidelines recommendation system	All suspicious lesions in high-risk groups should be biopsied and/or excised (D) Female patient with AIN should also be screened for gynaecological intraepithelial neoplasias (D)	AIN 2 and AIN 3 should be discussed and managed within a specialist anal cancer MDT (D) Consider HIV testing in persistent or multifocal anal dysplasia (C) For HIV positive homosexual men electrocautery may be better tolerated than topical treatments such as imiquimod and 5-Fluorouracil (B)	High-risk patients follow-up 6 monthly for 5 years (D)
Europe				
Italian Society of Colorectal Surgery (SICCR)	Binda et al. [27] Oxford Level of Evidence and US Preventive Series Task Force Grading Recommendations	High resolution and biopsy for histology a better test than anal cytology (2B) Digital rectal exam must be performed as well as anoscopy and anal cytology (1B) Genotyping HPV is not required if High resolution anoscopy and biopsies of concerning areas is undertaken (2B)	High-grade dysplasia should be treated either with topical treatment or ablative therapies (1B) Electrocautery recommended for anal canal lesions and Imiquimod for anal margin lesions (1B) Recommends the use of Imiquimod, 5-Fluorouracil, Cidofovir (2B), Photodynamic therapy and Trichloroacetic acid (2C), infrared coagulation, electrocautery, and carbon dioxide laser (2B) for the treatment of anal dysplasia Can also consider wide local excision of a lesion if less than 1/3 anal circumference (2B) Recommends vaccination for primary prevention of HPV elated dysplasias (1B) and HPV vaccination as secondary prevention for patients with previously treated anal dysplasia (3C)	Digital rectal examination, anal cytology, and high resolution anoscopy every 4 months for 3 years then annual follow – up for 2–3 years (2B) Follow-up at 6 and 12 months (2B)
German Society of Dermatology (GSD)	Esser et al. [28] GRADE Recommendation System	Annual screening recommended for HIV positive patients (1C) ^a^ Screening should include history and examination and anal cytology. If abnormalities patients should be referred for high resolution anoscopy (1C) ^a^ Other high-risk patients are defined as patients with a history of a previous HPV related dysplasias or cancers (including oral, anal and genital) and patients with a persistent HPV infection lasting longer than 1 year or condylomata acuminata. They should receive high resolution anoscopy once every 3 years. (1C) ^a^	Low- and high-grade dysplasia can be treated with topical treatments or surgical excision. (1C)^a^ The authors prefer surgical excision and ablative therapies over topical and recommend topical treatments as an adjunct therapy. (opinion) AIN 1 can be observed rather than treated if appropriate. (opinion)	Low- and high- dysplasia to be followed up in 3 months with high resolution anoscopy, cytology and clinical assessment (opinion) Can return to normal surveillance programme if four negative results 3–6 months apart (opinion)
European AIDS Clinical Society (EACS)	European AIDS Clinical Society Panel (2020) [29] GRADE Recommendation System	Screening recommended for HIV positive men who have receptive anal intercourse and HIV positive patients with a previous diagnosis of HPV dysplasia Digital rectal examination and anal cytology every 1–3 years in patients with HIV (1C)^a^ Anoscopy if anal cytology is abnormal (1B)^a^ Cervical screening in women who are HIV positive (1A)^a^	Efficacy of 9 valent HPV vaccine is questionable if HPV related dysplasias have already occurred (1A)^a^	No recommendations
Hellenic Society of Medical Oncology (HESMO)	Gouvas et al. [30] Oxford Level of Evidence and US Preventive Series Task Force Grading Recommendations	Screening recommended in high-risk populations (defined as HIV positive patients, MSM, women with history of cervical cancer, perianal condylomata and solid organ transplant recipients) (4B) Recommends annual digital rectal examination and anal cytology or if available high resolution anoscopy (4B)	Topical and ablative therapies acceptable for treating high-grade disease (3B)	Regular follow-up of high-grade disease is mandatory (A)

^a^Recommendation not specified in paper therefore GRADE Recommendations used by authors[1]

### Current guideline recommendations

#### Screening for AIN

Cytology or Papanicolaou smear tests have long had a role in cervical screening programmes with excellent sensitivity and specificity for the diagnosis of cervical intraepithelial neoplasias and cervical cancers [18]. In the United Kingdom, since cervical screening programmes were established the incidence of cervical cancer has decreased by 26% [19].

As ASCC is thought to have a similar natural history to cervical cancer anal cytology is a natural candidate for screening programme for AIN and ASCC. However, the literature is less convincing about its accuracy in its role for screening for AIN and ASCC with studies only an 83% sensitivity and 38% specificity [20] and high false negative rates.

Testing for oncogenic HPV genotypes has also been put forward as an adjunct to anal cytology screening [21]. There is little consensus about this in the literature, some studies showing that, unlike in cervical screening, HPV genotyping does not improve sensitivity or specificity of cytology [20]. When studying high-risk populations, many patients with high-risk HPV genotypes have multiple infections that develop, regress and clear spontaneously after exposure. It is likely that it is the chronicity of a high-risk HPV infection, rather than the acute exposure, that increases the risk of dysplastic change. Therefore, unfortunately, HPV genotyping at single points of time is unlikely to be beneficial.

Alterative screening methods include regular *per rectal* examinations as well as, what is many clinicians believe is the gold standard method, High Resolution Anoscopy (HRA). HRA however, is expensive and operator dependant with high-grade AIN and ASCC detection rates closely related to anoscopists experience [22]. However, when this technique has been compared to expectant management, the results have been disappointing and HRA was not shown to significantly affect detection rates or prevent progression of ASCC [23].

With this is mind, it is understandable that different regions have different recommendations about screening.

All regions apart from the UK named cytology as a screening tool but each region deals with cytology’s poor specificity differently, European AIDS Clinical Society (EACS), New York State Department of Health (NYSDOH) and the Hellenic Society of Medical Oncology (HESMO) recommend the use of cytology as a first line screening tool for all HIV positive patients advocating that only patients with an abnormality of cytology or patients who also have specific risk factors should then be referred for HRA. However, the American Society of Colon and Rectal Surgeons (ASCRS) guidelines recommends that it should only be used as part of a comprehensive screening programme and not as a first line tool alone.

The Italian Society of Colorectal Surgeons (SICCR) guidelines simply state that high resolution anoscopy (HRA) is a better screening tool. Whereas in the UK and USA HRA is recommended but only in the hands of experienced practitioners. The EACS and the NYSDOH guidelines also detail the timing of HRA screening in high-risk groups but differ in the best timings for this to take place. As there is insufficient information in the literature about screening timings, all the other guidelines recommend screening in high-risk groups to take place but stop short of stating clearly when or how to best go about this.

SICCR, NYSDOH and ASCRS give recommendations about HPV genotyping, the former advises that is not required if HRA and biopsies are taken whereas the ASCRS recommends use of HPV genotyping as an adjunct to an established screening programme. NYSDOH does not give recommendations about its inclusion or exclusion into a screening programme but does state that any patients identified with high-risk HPV genotypes should undergo HRA.

**Fig. 1:**
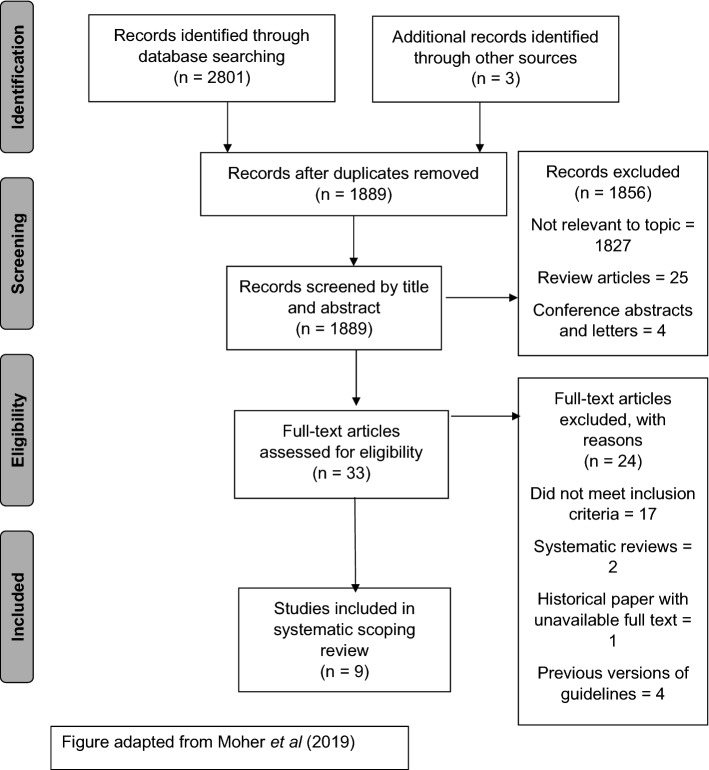
PRISMA Table [24]—results of the search strategy and reasons for exclusion

It is encouraging that all the current guidelines, with the exception of the UK ACPGBI and the NCCN guidelines now refer to AIN using the correct LAST criteria guidelines [13] (LSIL and HSIL) however, only the Italian SICCR and the American ASCRS directly refer to the use of p16 to differentiate high-grade lesions from equivocal low-grade lesions despite good evidence that this is beneficial [11–13] (Fig. 1).

#### Treatment of AIN

There are many different treatment modalities that have been put forward as a candidate to treat AIN. They include topical treatments such as Imiquimod and 5-Fluorouracil, ablative therapies such as infrared coagulation, laser treatment and electrocautery and surgical excision of the affected areas.

If we continue to liken AIN to cervical intraepithelial neoplasia, it is likely that by treating AIN it is possible to prevent progression to ASCC. This is the basis of the success of the cervical screening programme. However, like screening, this has not been entirely straightforward to demonstrate in the management of AIN.

A systematic review of the different available treatment modalities was recently undertaken and it found that although all the treatments did regress high-grade AIN, the recurrence rates were high and follow-up times were not long enough to demonstrate long term clinical benefit or the prevention of ASCC [31].

The advice in all included clinical guidelines reflects this equipoise. Some geographical regions are much more proactive about treating AIN as they view the potential risk of ASCC higher than the potential risk of the treatment. For example, the Italian SICCR guidelines state the opinion that it is unacceptable to not treat AIN and state that all patients should be treated. Similarly, the NYSDOH guidelines recommend in their subset of patients, AIN2 and AIN3 should be treated whereas AIN 1 can be observed.

However, clinical guidelines from the UK, Germany, and ASCRS state you can treat AIN but place less emphasis on this advice. They also do not recommend which treatment, if treatment is undertaken, is recommended practice. Rather, in the UK ACPGBI guidelines, they stress the importance of the use of specialist multidisciplinary team meetings to permit best practice in a grey area of clinical benefit.

Other guidelines are less vague; the ASCRS guidelines limit ablative therapies to the treatment of high-grade AIN. Also, based on a randomised controlled trial [32] comparing electrocautery to Imiquimod and 5-Fluorouracil the ACPGBI and National Comprehensive Cancer Network (NCCN) guidelines recommend that electrocautery may be better tolerated than topical treatment in HIV positive men who have sex with men.

#### Follow-up of AIN

As the benefits of treating AIN are unclear so are the procedures for follow-up after treatment for AIN. Unfortunately, there are not enough studies in the literature that have followed up patients long enough to be able to identify an ideal follow-up protocol [31].

The rate of progression from high-grade AIN to ASCC varies considerably in the literature with some studies quoting up to a third of patients developing ASCC 3 years after diagnosis of high-grade AIN [33]. Lee et al. demonstrates a median time of progression of 2.7 years [34] and Scholefield et al. states the risk of progression to ASCC from AIN is 10% at 5 years [35]. However other studies exist that suggest that transformation of AIN to ASCC is a much rarer occurrence with 7.4 cases per 100 person years of follow-up [36].

All guidelines, apart from the ASCRS guidelines who state that treated patients do not require follow-up unless visible or palpable disease develops, recommend some regime of follow-up in patients with AIN. Most guidelines recommend between 3–6 monthly follow-up for 3–5 years, however the NYSDOH guidelines recommend also yearly HRA follow-up for AIN1.

#### Recommendations for immunosuppressed and transplant patients

One paper was found that discussed the screening for and treatment of HPV infections in patients with solid organ transplantations. The American Society of Transplantation (ASOT) gives recommendations that all transplant recipients who have a history of receptive anal intercourse or cervical dysplasia should undergo anal cytology screening, furthermore they recommend high-grade disease should be treated whereas low-grade dysplasia can be observed. They do not give any recommendations on the treatment modality that can be used nor do they specify timings for screening or low-grade observation [37]. There were no papers identified in the scoping review that discussed the management of patients with other causes of immunosuppression.

## Evaluation of preceding guidelines in comparison to current clinical guidelines

In our literature review, four further clinical guidelines were identified that had been superseded by more recent up to date clinical guidelines, two of these guidelines were from Europe (German Society of Dermatology and SICCR) [38, 39], one from the USA (ASCRS) [40] and one from the United Kingdom (ACPGBI) [35]. When evaluating each included guideline with its preceding counterpart it is clear to see there has been a definite drift from recommendations from as little as 5 years ago advocating a much more aggressive approach now to the management of AIN.

The ACPGBI guidelines in 2011 did not advocate screening for AIN, nor did it recommend treatment or long-term follow-up of patients with low-grade AIN [35]. Interestingly, both the historical ASCRS and the SICCR guidelines recommended anal cytology for AIN screening rather than HRA [38, 40].

The historical ASCRS guidelines also have subtly changed their wording from patients can be observed in select cases to patients can be treated [40]. There has also been a change in the principles of treating AIN, previous guidelines in the UK and Germany particularly emphasise the excision of dysplastic areas as a definitive treatment for high-grade AIN. However this is largely now fallen out of favour due to the risk of anal stenosis and the high rates of recurrence in case series [41].

Anal mapping, a mainstay of AIN surveillance in the UK previously has now been discouraged in the recent UK guidelines due to futility and risk of patient harm [6].

The historical guidelines recommend considering HPV vaccination in high-risk groups after some success in the secondary prevention of AIN in a study in 2012 [42]. However, in 2018 a randomised controlled trial using the quadrivalent HPV vaccination as secondary prevention for patients treated for AIN did not demonstrate any benefit therefore this recommendation is no longer in the most up to date guidelines [43].

The historical and current SICCR guidelines are relatively similar to each other and the most consistent over time, however, they also are the most enthusiastic about treating AIN overall.

Prior to the completion of this systematic scoping review, it was believed that different geographical areas have different recommendations for the screening, treatment and follow-up of AIN but that there was local consensus within a geographical area. This was thought to be related to different healthcare systems having different access to treatments and screening practices and the availability of local clinician expertise. Although the historical guidelines largely follow this pattern, this effect is less apparent in the current clinical guidelines available.

## Discussion

It is interesting that the guidelines are starting to drift towards some agreement about the best practice in following up patients with AIN despite the lack of evidence base. It also appears that it has become less acceptable to just observe high-risk patients.

However, there does not seem to be much change in the lack of consensus in screening high-risk patients despite all guidelines now reporting a need to identify them. Unfortunately, there is not a screening technique that does not have significant flaws, anal cytology has a low sensitivity and high false negative rate, HRA is expensive and inaccurate unless used by expert practitioners, serial *per rectal* examinations in high-risk patients is also significantly dependant on clinicians experience and thorough note keeping. Nevertheless, when used by expert practitioners, HRA guided biopsies are the gold standard in AIN screening and are the most likely to be diagnostic [27]. Isolated HPV genotyping is potentially flawed in high-risk populations as they are more likely to have multiple HPV infections that develop, regress and clear spontaneously after exposure. It is likely that it is the chronicity of a high-risk HPV infection, rather than the acute exposure, that increases the risk of dysplastic change. Identifying those patients who do not clear the infection is potentially a better indicator of risk.

The inclusion criteria of this scoping review allowed for the inclusion of all clinical guidelines where recommendations are given on the screening, treatment, and follow-up of AIN. The rationale for this was that AIN is a subspecialist topic and guidelines often treat AIN as a subtopic of ASCC including recommendations for AIN within a section of larger ASCC guidelines. Indeed, of the nine guidelines included in this scoping review only four papers identified were guidelines solely related to the care of AIN [26, 27, 29, 37], and only one of these papers was about the management of AIN overall [27] rather than in a subgroup of people such as patients with solid organ transplants or patients with HIV. Although all guidelines were endorsed by clinical societies, guidelines written where AIN is included as a subsection of other guidelines may not have the detailed overview of the literature as much as guidelines dedicated to AIN.

The increasing awareness of high-risk patients and the potential benefits of HRA is to be credited to specialised societies such as the International Anal Neoplasia Society who advocate for this specialised patient subgroup and provide HRA training. However, until the treatment of AIN becomes more mainstream and larger scale study results are available the guidance of how best to manage these patients may not improve. The challenge remains in identifying the patients most at need of expert care.

It is also important to note that the literature is heavily weighted towards HIV positive men who have sex with men (MSM) and there is a lack of clinical study into HIV negative women with AIN despite women having the highest incidence of ASCC [44] as such the clinical guidelines often appear to underestimate their risk when compared to HIV positive MSM. Indeed, the majority of clinical studies involving the treatment, surveillance and follow-up of AIN exclusively include HIV MSM as they are the most easily available and recruitable patients in this subspeciality. As such, there is little evidence on the management of patients who are not HIV positive men which significantly limits the scope of guidance available.

Like a previous systematic review [45] comparing historical clinical guidelines there as significant discrepancy on the terminology of AIN between geographical regions and in particular the use of AIN2 as low and high grade. This limited generalisability between recommendations. To prevent confusion, we continued using the nomenclature used by guidelines in the results table.

This systematic scoping review is limited by the restriction of included papers to guidelines written in the English language. Although both reviewers did not identify any guidelines in any other language it is possible that guidelines written in areas of the world with high prevalence of ASCC, for example Brazil and Russia, have their own guidelines that could not be included in this paper. Also, the authors were surprised that countries such as Australia and New Zealand did not have accessible guidelines this could potentially bias this review and its findings that there is an increasing trend to treat AIN. Clinicians in Australia in particular are associated with a trend for more conservative management in expert conferences when compared to their American counterparts.

The comparison of strength of recommendations between guidelines is a study limitation and a potential source of confusion for the reader, as different societies had preferences for different grading systems yet still use similar nomenclature. The authors recommend that readers use care to identify the source of the grading system identified in Table 1 despite the nomenclature being similar IC in Oxford Levels of Evidence has a considerably different meaning to 1C in a paper using the GRADE recommendations.

## Authors’ recommendations

Despite the lack of available evidence base, we feel that the correlation of data suggests an agreement in some of the issues raised. As such we would recommend the use of HRA by expert practitioners as the screening method of choice for high-risk individuals in particular HIV positive men who practice receptive anal intercourse and patients with previous AIN or HPV perineal dysplasias (1C). Arguably, despite the American Society of Transplantation recommending anal cytology as a first line screening tool in solid organ transplant recipients, solid organ transplant recipients should be included in this high-risk group where HRA is offered first line by expert practitioners. Anal cytology can be performed at the same time as HRA as an adjunct for diagnosis but should not be the main screening tool. For patients with lower risk factors, for example women with HIV and men with HIV who do not practice receptive anal intercourse (medium-risk individuals), on the balance of risks anal cytology is likely to be the best screening method as this could be delivered routinely without input from expert practitioners. There is no correlation in guidelines currently with the timings of screening programme for high-risk and medium-risk patients. Nevertheless, given that the median progression rate of high-grade AIN to ASCC is quoted as 2.7 years [34] or a third of patients three years after high-grade AIN diagnosis [33] annual examinations in high-risk individuals would not be unreasonable and medium risk patients, without high-grade disease could have longer times between screening attendances.

All high-grade AIN (HSIL, HGAIN and AIN3) in HIV positive men who have receptive anal intercourse, on the balance, should be treated. There is some evidence that electrocautery may be the best choice of treatment in these patients (1B) [32]. As there is a paucity of research yet on whether treating AIN is beneficial to patients who are not HIV positive men who practice receptive anal intercourse, it is unclear whether treating high-grade AIN in patients in subgroups with different risk factors is beneficial without further research.

All except one guideline recommend following up treated high-grade AIN every 3–6 months for between 3–5 years.

## Conclusion

When evaluating clinical guidelines giving recommendations on the screening, treatment and follow-up of patients with AIN there appears to be increasing consensus on the treatment and follow-up of patients despite a poor evidence base. Nearly all included guidelines recommend the treatment and follow-up of high-grade AIN, but most stop short of recommending a specific treatment modality. There is still significant discrepancy in guidance on the method to identify patients at risk of ASCC and AIN despite consensus between geographical regions on which patient subgroups are at the highest risk.

## Data Availability

Not applicable.
